# Using symbiotic empirical ethics to explore the significance of relationships to clinical ethics: findings from the Reset Ethics research project

**DOI:** 10.1186/s12910-024-01053-9

**Published:** 2024-05-28

**Authors:** Caroline A. B. Redhead, Lucy Frith, Anna Chiumento, Sara Fovargue, Heather Draper

**Affiliations:** 1 Centre for Social Ethics and Policy, Department of Law, The University of Manchester, Manchester, UK; 2 School of Social and Political Sciences, University of Edinburgh, Edinburgh, UK; 3 School of Law, University of Sheffield, Sheffield, UK; 4 Warwick Medical School, The University of Warwick, Warwick, UK

**Keywords:** Covid-19, Bioethics, Empirical ethics, Maternity, Paediatrics, Qualitative research

## Abstract

**Background:**

At the beginning of the coronavirus (Covid-19) pandemic, many non-Covid healthcare services were suspended. In April 2020, the Department of Health in England mandated that non-Covid services should resume, alongside the continuing pandemic response. This ‘resetting’ of healthcare services created a unique context in which it became critical to consider how ethical considerations did (and should) underpin decisions about integrating infection control measures into routine healthcare practices. We draw on data collected as part of the ‘NHS Reset Ethics’ project, which explored the everyday ethical challenges of resetting England’s NHS maternity and paediatrics services during the pandemic.

**Methods:**

Healthcare professionals and members of the public participated in interviews and focus group discussions. The qualitative methods are reported in detail elsewhere. The focus of this article is our use of Frith’s symbiotic empirical ethics methodology to work from our empirical findings towards the normative suggestion that clinical ethics should explicitly attend to the importance of relationships in clinical practice. This methodology uses a five-step approach to refine and develop ethical theory based on a naturalist account of ethics that sees practice and theory as symbiotically related.

**Results:**

The Reset project data showed that changed working practices caused ethical challenges for healthcare professionals, and that infection prevention and control measures represented harmful barriers to the experience of receiving and offering care. For healthcare professionals, offering care as part of a relational interaction was an ethically important dimension of healthcare delivery.

**Conclusions:**

Our findings suggest that foregrounding the importance of relationships across a hospital community will better promote the ethically important multi-directional expression of caring between healthcare professionals, patients, and their families. We offer two suggestions for making progress towards such a relational approach. First, that there is a change of emphasis in clinical ethics practice to explicitly acknowledge the importance of the relationships (including with their healthcare team) within which the patient is held. Second, that organisational decision-making should take into account the moral significance afforded to caring relationships by healthcare professionals, and the role such relationships can play in the negotiation of ethical challenges.

**Supplementary Information:**

The online version contains supplementary material available at 10.1186/s12910-024-01053-9.

## Introduction

The response to the coronavirus (Covid-19) pandemic has had far-reaching consequences for the National Health Services (NHS) in the UK, including posing challenges to many of the principles and presuppositions informing the clinical ethics frameworks by reference to which healthcare professionals (HCPs) offer care to patients and their families. In choosing how to ‘reset’ non-Covid-19 healthcare services alongside a continuing pandemic response,^1^ healthcare decision-makers had to consider how ethical considerations did (and should) underpin decisions about integrating infection prevention and control (IPC) measures into routine healthcare practice. New kinds of ethical issues and dilemmas arose as assessments were made as to how best to balance patients’ and families’ access to healthcare services with the protection of both hospital communities and the wider public from Covid-19. Guidelines and policies, rapidly developed to underpin the acute coronavirus response, used pandemic-specific approaches to decision-making in anticipated worst-case scenarios [1]. These frameworks, however, demonstrated little attention to the challenges inherent in balancing pandemic responses with the concurrent provision of *non-pandemic* healthcare.

The ethical challenges of (re)organising healthcare services to facilitate the provision of maternity and paediatric services during Covid-19 was the focus of the multi-disciplinary Reset Ethics project (Reset).^2^ Maternity services were chosen because they could not be suspended, and paediatric services as children were at lower risk of severe effects of Covid-19 infection [2]. In addition, professional and patient organisations were highlighting the adverse impacts of efforts to balance these services with measures to respond to Covid-19 [3]. Further, our rapid review of ethical values guiding decision making in resetting non-Covid-19 paediatric and maternity services had indicated the potential impact of IPC decisions, such as visiting restrictions or virtual care, on services where family-oriented, relational care is usually embedded in service structures and models of delivery [1]. Our central aim was to consider how decision-makers understood and attended to the ethical issues and dilemmas arising in the context of ‘resetting’ non-Covid services alongside the continuing pandemic response after the UK Government’s announcement on 29th April 2020.^1^We had anticipated that services would be ‘reset’ during a time-limited ‘reset period’ within which specific IPC measures were mandated. However, as the various phases of the pandemic continued, services were continually re-adjusted to respond to various mandatory measures to limit the spread of infection, from ‘lockdowns’ and other restrictions on social interaction, to measures specific to clinical settings, such as visitor restrictions and the wearing of personal protective equipment.^3^ Our qualitative data, collected between November 2020 and July 2021, indicate that significant challenges were encountered by HCPs in their struggle to comply with (sometimes rapidly changing) IPC measures and, at the same time, offer the level of patient care they felt their personal standards and professional obligations required. The mandating of personal protective equipment (PPE), the social distancing requirements and the measures imposed to reduce footfall within hospitals (such as banning birth partners and allowing only one parent at a time to be with a hospitalised child) were experienced by HCPs as barriers to their engagement with patients and their families; barriers which impeded on the creation and development of supportive relationships. Our data indicate that, for HCPs, offering care as part of a relational interaction is an ethically important dimension of healthcare delivery [4].

In this article, building on our findings, we explore the significance of relationships to clinical ethics, with the aim of using our empirical data to generate possible solutions for the ethical challenges described by the HCPs who participated in our research. We describe our use of an empirical ethics methodology to work from our empirical findings towards the normative suggestion that, particularly in maternity and paediatric services, clinical ethics should explicitly attend to the importance of relationships in clinical practice. Our article thus makes a contribution to debates over how clinical ethics should be conceptualised, and how current models could be improved. Our suggestions could have general application to all clinical practice, and not just to practice in pandemics or emergency situations.

The article proceeds as follows. We start with an introduction to the Reset research, describing the background, aims and objectives of the project. We then offer some context for the discussion of our findings, summarising the conceptual underpinnings of clinical ethics, to provide context for our claim that relationships are not prioritised (although they are starting to be recognised) in current clinical ethics. We next situate that claim in a brief theoretical discussion of the notion of relationality, particularly in a healthcare context. A discussion of the theoretical and methodological aims of empirical bioethics follows. Empirical bioethics is a method of integrating empirical research and normative enquiry, and we use it to bring together our qualitative data and the ethical questions we have considered in the research setting. This discussion provides a methodological context for the introduction of Frith’s symbiotic empirical ethics methodology, a particular approach to empirical bioethics, to which we then turn. This methodology informs the development of the thematic analysis of our qualitative data.

Using Frith’s method, we explore the ethical issues experienced during the pandemic by our participants. Working backwards and forward between our qualitative data and the normative issues identified, we examine the disjunct identified by our participants between the patient-centred focus of clinical ethics and the broader community safety-based concerns of public health ethics. It was the experience of this collision between what we will call everyday clinical ethics, ie., pre-pandemic clinical ethics, and pandemic ethics based on public health ethics, thrown into sharp relief in the ‘resetting’ of healthcare services during the pandemic, which was challenging for the HCPs who participated in our research. We conclude with the normative suggestion that, in its conception of ‘the patient’, clinical ethics should explicitly acknowledge the importance of the relationships within which the patient is held (including with their healthcare team) and, in so doing, better (and explicitly) reflect the importance of relationality in healthcare. This shift in framing requires an acceptance of the claim made by relational theorists that, rather than existing as autonomous individuals in the Cartesian sense, it is within and by their ‘*networks of relationships*’ that people are constituted (emphasis added) [5].

## Background

### The Reset Ethics research

There is a large body of literature on ethical frameworks and discussion of national and international ethics frameworks for pandemics [6], but little empirical research on how such frameworks are applied and utilised in clinical practice. The pandemic injected urgency into these questions, and focused attention on the need to know how to support hospital managers and HCPs making ethical decisions, often at pace, in these unfamiliar circumstances. Insufficient consideration was given, however, to the policies and guidelines put in place to manage the kinds of ethical issues and dilemmas that were arising due, for example, to the nationally mandated IPC measures that were rapidly brought in to support the initial response to the pandemic and the changes in working practices that resulted [7]. In particular, there had been little or no ethical assessment of the policies whose aim was to re-organise clinical services [8].

The Reset project thus identified, and responded to, an urgent need to evaluate and support ethical clinical decision-making in maternity and paediatrics services. We did not work to answer a specific research question but, rather towards a broad aim to examine the everyday ethical challenges of re-organising these services during the pandemic, to understand how HCPs had interpreted and employed the resulting policies and decision-making processes in practice, and to identify what ethical issues had caused concern in their clinical practice. Our objective was to suggest mechanisms for ethics support for HCPs working within the constraints of pandemic-related changes to service provision, by providing empirically-based, accessible and feasible recommendations for embedded ethics support in policy and clinical practice [9]. Ultimately, aim of the project was to make a contribution to normative theory on the basis of empirical data. To do this, we employed the symbiotic empirical ethics approach [10], where philosophical theory is used to explore empirical data, draw normative conclusions, and make policy and practice recommendations. The empirical work has been reported elsewhere [4] and the focus of this article is our use of Frith’s empirical bioethics methodology, symbiotic empirical ethics (a detailed introduction to which is offered below), to generate normative solutions to the challenges experienced by the HCPs who participated in our research. We specifically consider whether we should go back to clinical ethics’ previous focus on the atomistic individual patient, or whether a re-adjustment should be made so that everyday clinical ethics practice acknowledges, by paying attention to ‘the patient-in-relationships’, that people are ‘in basic ways constituted by the networks of relationships of which they are a part’ [5].

### Clinical ethics and public health ethics

We will define clinical ethics broadly to include the ethics that guide all HCPs in their interaction with patients. At the core of clinical ethics is an emphasis on the relationship between an HCP and a patient in the clinical setting [11]. The relationship is a one-to-one relationship – premised on a clinician’s professional obligation to make the care of their patient their first concern [12]. The roots of this individual focus of a clinician’s professional attention lie in the Greek medicine tradition, reflected particularly in the Hippocratic Oath, which remains the basis of the (current) World Medical Association’s Declaration of Geneva, where similar wording appears [13]. Furthermore, in clinical ethics the doctor-patient relationship is centred on an understanding of patients as rational, self-interested, transparent individuals, guided by their conscious personal wishes, making decisions for and by themselves [14]. Clinical ethics is thus predominantly focused on a HCP-patient dyad, with the ethical course of action aligned, as far as possible, with the preferences and values of the individual (autonomous) patient [15].

Public health ethics, by contrast, flow from the state’s duties to protect all citizens from harm, including, to the extent possible, from infection with transmissible diseases [16]. The focus is thus on population-level duties to promote equality of persons and equity in distribution of risks and benefits in society. In the circumstances of a pandemic, government action is intended to protect the community, even where (temporary) curtailments of individual rights result – the decision to impose nationwide ‘lockdowns,’ during the Covid-19 pandemic, for example. For HCPs, there can be tensions associated with the shift from the individual-patient-centred practice of clinical ethics to patient care guided by public health considerations. These tensions, however, are not new. In practice, even in ‘usual’ circumstances, HCPs are rarely in a position to focus only on the care of one patient, because to do so would often act to the detriment of others under their care or supervision. Thus, as Daniel Sokol has recently compellingly described, HCPs always have to prioritise some patients over others [17]. In the National Health Service (NHS), which has for some years been stretched, the pandemic has thus exposed and magnified existing tensions between the individual focus of clinical ethics and broader public-health concerns.

The importance of balancing clinical ethics and broader public health concerns is also reflected in professional guidance. For example, doctors practising in the UK are required to comply with the General Medical Council’s (GMC) guidance for good medical practice [12]. This guidance is split into four sections which together describe and proscribe the professional values and behaviours expected of registered clinicians. The GMC guidance current at the time of writing, required doctors to make the care of their patient their first concern, reflecting the individual focus described above.^4^ However, in addition to making the care of their patient their first concern, doctors following that guidance were also expected to protect and promote the health of patients *and* the public [18]. Similarly, the most recent version of the Declaration of Geneva asks physicians to pledge to share their medical knowledge for the benefit of the patient *and* for the advancement of healthcare, and, in addition, requires them to attend to their own health, well-being, and abilities, in order to provide care of the highest standard [13]. Graeme Laurie, Shawn Harmon and Edward Dove describe these additions to the Declaration of Geneva as ‘a more communitarian turn in the tenor of the document’ [19].

A new version of the GMC’s guidance came into effect in January, 2024. Small but potentially significant changes to the way doctors’ responsibilities are framed have been made, such as, for example, a requirement to make the care of *patients* their first concern [20]. Contrasting this wording to the previous requirement (to ‘make the care of *your patient* your first concern’ (emphasis added)) imports into the guidance an acknowledgement of broader, population-level issues. The current guidance for nurses, midwives and nursing associates is drafted similarly, requiring them to put the interests of people using or needing nursing or midwifery services first [21]. Thus, it is clear that, in offering care, HCPs are expected to consider more than just the individual patient in front of them and, thus, that the wider community is (becoming) an important part of clinical ethics.

Certainly, the current versions of the GMC Guidance, the Nursing & Midwifery Council Code and the Declaration of Geneva ask HCPs to *look beyond* the needs of the individual patient in front of them and require them, in doing so, to use their professional judgement and expertise to interpret and apply these principles to the various situations they face. Each individual HCP is thus required to assess what they consider to be an appropriate balance between their duty to an individual patient as their first concern and their (potentially conflicting) duty to the wider community. However, mandatory pandemic IPC measures often left no room for the exercise of professional discretion in balancing duties to individual patients with the protection of the wider hospital community. During the early, acute stage of the pandemic, when SARS-CoV-2 was emergent and poorly understood, the need for strict enforcement of IPC measures was clear. However, as the pandemic progressed and understanding of its risks grew, HCPs working in maternity and paediatric settings felt that a different approach infection prevention was sometimes needed to recognise the importance to patients and their families of human contact. This was the starting point for many of the ethical challenges our participants described.

We argue below that the experiences of Covid-19 have brought into sharp relief the need to explicitly recognise the importance of wider relationships to clinical ethics. Rather than limiting its concern to the individual patient, albeit acknowledging the concomitant importance of the health of the general public, we suggest that clinical ethics needs also to attend to the importance of the relationships within which people live, and to bring patients’ family members and friends into the scope of its consideration. Although each patient’s supporting relationships could arguably be included in the requirement to make their care a HCP’s first concern, our data suggest that an *explicit* recognition of the *value* of relationships in clinical care is needed. This means a shift must be made in how we prioritise ethical values, away from the ‘usual’ patient-centred framing and towards a more distinctive relationships-based integration of clinical and public health ethics, which acknowledges the constitutive relationships within which patients live. To explore this further, we will now consider theories of relationality and briefly discuss the significance of a relational perspective to clinical ethics and the practice of healthcare. We will build on this theoretical foundation as the article progresses.

### Relationships and theories of relationality

In the healthcare context, patients live not ‘in a cocoon’ but in a relational, social and cultural environment that conditions and limits their healthcare decision-making [14]. This was starkly evident during the COVID-19 crisis, when many families suffered because they were unable to visit loved ones or, worse, to be with loved ones as they died. Carlos Gómez‑Vírseda and Rafael Usanos, reflecting on the legacy of the pandemic, have argued that relationality should be ‘integrated into the heart of bioethical theory’ [14]. Charting the development of philosophical accounts of relationality in the social sciences (the philosophical branches of cultural anthropology, philosophy of nature, and discourse ethics), and the evolution of phenomenology (philosophical anthropology, existential phenomenology and hermeneutics), Gómez‑Vírseda and Usanos distil several key insights into the relational model of autonomy they suggest [14]. First, that the biocultural human being exists within relations to and with other humans, which means that cultural and social relationships are fundamental to human existence [22]. Second, from a cosmological perspective, everything is connected and so humans and human societies are one aspect of an interconnected world, within which everything is (inter)related [23]. Third, these (inter)relationships, at both a human and a global level, have consequences for ethics. Gómez‑Vírseda and Usanos, using earlier philosophers’ accounts of relationality, describe how the solitary moral conscience has given way to a discursive ethics approach, such that the reasoned agreement of everyone affected by a normative system is essential to its validity [24].

Gómez‑Vírseda and Usanos set out what they term the ‘bioethical implications’ for each philosophical development they map. While their article seeks to offer a reasoned justification for a relational model of autonomy, their broader discussion about relationality and bioethics speaks to our contention here that an explicitly (more) relational approach to *clinical ethics* is warranted, for the benefit of HCPs and patients *and* their loved ones. As Sokol has described, and our data confirm, such an approach would offer fairer reflection of real clinical practice [17]. The relational environment, which involves patients’ family, friends and communities, and, of course, the team(s) of HCPs offering care to the patient, inevitably conditions the way care is experienced and the way treatment decisions are discussed, understood and agreed. In addition, a recognition of the relational character of the bioethical subject facilitates dialogue with moral traditions with different starting points from those which underpin Anglo-American clinical ethics. The inclusive African knowledge system, *Igwebuike*, is an example of such a tradition [25]. Underpinned by the metaphysical assumption that the world is a totality of interconnected and interrelated entities, *Igwebuike* (which literally translates as ‘number is strength’ [25]) accepts the moral relatedness of all humans. Notions of solidarity, companionship and identification with others are considered to be virtues that promote human flourishing. Actions that diminish ‘humanness’ are explicitly forbidden [26].

This idea of the importance of ‘humanness’ can clearly be seen in the Reset project data, where IPC measures were experienced by participants (HCPs, patients and their families) as barriers to the experience of giving and receiving compassionately ‘human’ care during the pandemic [4]. ‘Humanness’ in this sense can be understood as an important component of an understanding of relationality as an ethic of care, a feeling of being connected to other people and empathising with them [27]. The significance of the ‘face-to-face’ is clearly seen, with perceived barriers (such as face-masks) disrupting human connection and relationships [28]. Our research findings thus reconfirm the importance of these theoretical ideas in healthcare practice. As we argue below, it is important, for the well-being of patients, family members and HCPs, that clinical ethics attends *explicitly* to the importance of relationality in healthcare. Although the current Nursing & Midwifery Code and the new iteration of GMC’s Good Medical Practice^5^ make space for the relevance of a wider frame of reference for HCPs than just the patient in front of them, we argue that a further shift is needed, clearly and explicitly to understand each patient as part of a network of supportive relationships. Our research shows how important an acknowledgement of those relational networks can be for someone’s experience of healthcare. In order to provide some context for the use of our qualitative data to underpin our call for a clearer acknowledgement of relationality in clinical ethics, we will now summarise the theory and practice of empirical bioethics.

### Empirical bioethics: a summary

Empirical bioethics is a theoretically complex form of research [29]. In carrying out empirical bioethics research, those active in this field work through the methodological, empirical and metaethical challenges of integrating the empirical and the normative, often as part of interdisciplinary teams. The central aim of empirical ethics is to *integrate* these two elements of the research, rather than to conduct the empirical research and the normative enquiry in parallel, or to use empirical data solely a means to an end. In order to do this, clarity is required about how (and why) the empirical data inform normative conclusions and why (and how) those normative conclusions are justifiable. Thus, the key challenge for empirical bioethics research is to achieve coherence in moving from empirical data describing how things *are*, to normative claims about how those things *ought* to be [30]. Jonathan Ives and Heather Draper have suggested that this requires ‘respect[ing] the sound empirical point that facts and values are not distinct in practice … [and] not falling foul of the is/ought problem as defined in philosophical terms’ [31].

A variety of methodologies has been proposed to bridge the gap between the empirical ‘is’ and the normative ‘ought’ [32]. In their systematic review of 33 publications containing 33 distinct methodologies, Rachel Davies and colleagues concluded that the majority of the methodologies could be classified as either dialogical or consultative, or a combination of the two [32]. They described the aim of dialogical approaches as the co-construction of a shared understanding (usually of a particular problem) through dialogue between researchers and participants. Consultative approaches, by contrast, engage with participants through the collection of empirical data, which is then analysed separately by the researchers, who develop normative conclusions either to propose answers to particular problems or to develop ethical theory.

Our use of the symbiotic empirical ethics methodology to integrate our empirical findings with our normative enquiry is described and discussed in the following section.

## Methods^6^

Five NHS trusts took part in the Reset project,^7^ and were recruited during the autumn and winter of 2020, when hospitals in the UK were under significant strain as a result of the coronavirus [33]. We interviewed senior hospital managers and HCPs, and held focus groups with HCPs and members of the public. A full description of our data collection and analysis is provided elsewhere [4]. A timeline of the research activity, and selected demographic information, is provided in Table 1 below. Interview participants were asked to reflect on decision-making around how best to re-organise and reset services, and to consider the ethical implications of changes on HCP’s working practices. The ethical challenges described by the interview participants were then further explored in a series of focus groups with members of the public, hospital-based clinical psychologists, and HCPs. The topic guides for the semi-structured interviews and focus groups are included in Additional file 1.

**Table 1:** Research activity and timeline

Research activity	When activity carried out	Participant information	
Trust recruitment and set up	Permission to proceed: Trusts A/ B—October 2020 Trust C – November 2020 Trust D –January 2021 Trust E –June 2021 Trust F –February 2021		
Interviews: Senior Managers	November 2020 – June 2021	*N* = 11	female *n* = 7 male *n* = 4 clinical background *n* = 8 other professional background *n* = 3
Interviews: HCPs	January – July 2021	*N* = 26	female *n* = 20 male *n* = 6 doctors *n* = 9 nurses *n* = 12 midwives *n* = 5
Public focus groups [*n* = 5]	May 2021 (2 focus groups) June 2021 (3 focus groups)	*N* = 26	Focus groups had between 3–7 participants Female *n* = 24 Male *n* = 2 Purposive sampling of members of the public involved with maternity or paediatric surgery services since April 2020. Recruitment from hospitals throughout England, but focusing on NHS hospitals where interviews had been conducted. Participants identified attending *n *= 13 NHS Trusts
Clinical psychologist focus group [*n* = 1]	July 2021	*N* = 6	Female – *n* = 6
HCP Focus groups [*n* = 3]	1. August 2021 2. August 2021 3. September 2021	*N* = 7	Female – *n* = 7 Physiotherapy – neurology/neurosurgery *n* = 1 Physiotherapy – transplantation/oncology *n* = 1 Medical complexities *n* = 1 Paediatric trauma *n* = 1 Occupational therapy *n* = 2 Paediatric palliative care *n* = 1

The Reset study offers important insights into the experiences of NHS senior managers and HCPs working in paediatric and maternity services during the ‘reset’ phases of the pandemic, as well as the reflections of patients and family members who interacted with these services during an unprecedented period for the NHS. As services were ‘reset’, pandemic and ‘everyday’ health practices, and clinical and public health ethics were in tension in various ways. These tensions illuminated the experiences of our participants. Our research develops and extends the ‘Covid-19’ literature, adding an important dimension to complement studies that looked at ethical challenges arising in the acute phases of the pandemic. Some important limitations to our data must, however, be recognised. First, we recruited low numbers of participants from black and minoritised ethnic communities, despite their high representation in frontline NHS staff, and we struggled to reach more junior HCPs. We may also have missed the views and experiences of those shielding. Further, we used existing participation or patient involvement groups as routes to recruitment of our public focus group participants, which meant that many of them were active in hospital governance structures and/or patient involvement groups. This may have led to an emphasis on particular aspects of participants' experiences due to pre-existing knowledge of NHS systems. Finally, while our view is that all healthcare provision is underpinned by relationality, we note that the particular importance of relationships in maternity and paediatrics might limit the relevance of our findings to other areas of healthcare.

To draw normative conclusions from our data, we used a practical empirical bioethics methodology, Frith’s symbiotic empirical ethics, for approaching ethical questions in practice [10]. Classified by Davies and colleagues as a consultative methodology, but towards the dialogical end of the spectrum [32], this methodology consists of five elements: a description of the circumstances under consideration; a specification of the relevant ethical theories and principles; the use of ethical theory as an analytic tool; theory building; and, finally, the making of normative judgements [10].

## Results

We present our results using the staged methodology of symbiotic empirical ethics. We draw on the situated experiences of the HCPs and members of the public who participated in the Reset project to support our suggestion that a more clearly relational approach to clinical ethics would better support HCPs and patients, as well as patients’ families and their broader care networks, and, in so doing, enhance the wider aim of clinical ethics to ensure good quality care. To aid readability, the quotes reproduced in this article have been ‘cleaned up’ and shortened where appropriate.

### Setting out the circumstances

Our qualitative interviews with participating trusts’ senior decision-makers aimed to tease out the ethical values guiding the approach to decision-making and the justifications for the decisions made, whether implicit or explicit. Interviews and focus group discussions with HCPs sought to explore senior management decision-making from a different perspective. These interviews focussed on the way(s) participants’ working practices *had* to change to accommodate the resetting of paediatric and maternity services. We explored how participants felt about these changes and asked them to share any ethical challenges or difficulties that they had experienced as a result. Our data indicated that the ethical challenges encountered by HCPs were due to changes to their working practices mandated by pandemic IPC measures that impacted on all relationships within the hospital setting:

*But obviously once you restarted the services, you went in your bed space, you stayed in your bed space, they couldn’t you know…go and sit next to…[name’s] mum in bed eight because she’s upset because he’s going to theatre. It’s a very different feel. And we policed that quite strongly. So…we very much policed that there was no interaction between the families. And that’s not you know, we’ve grew up on a ward that’s very sociable, the kids will often play, the physios will get the two children throwing balls to each other across the bed spaces and you know, it’s quite a friendly Ward […] our Ward is quite a community feel, especially amongst the parents, and the staff will often look after the same patients for weeks so that there’s quite a relationship built up there. […] I’d quite often sit with [a] Mum for half an hour, because you’d be upset and you know, you’ve built up that relationship. Well obviously the more community side of the ward had to stop.* [Ward manager, trauma, orthopaedic, ENT and spinal unit. Interview participant.]

The impact on relationships was a key area of ethical concern for our participants because of the importance of the relationships within which maternity and children’s services are situated, and around which they are organised. These include relationships between HCPs and patients, between HCPs and patients’ families, between patients and their families, and between clinical teams:

*[T]he visiting policy across the hospital and restricting that to one parent, per patient, and at some periods of time, nobody, for a period of time as well, you know, really restricted… What staff saw, and the stress that with parents having to make some decisions on their own. And parents having to take young people to theatre where they might normally go together, not knowing whether their child would come back, I certainly had a mum that, I would never normally do this, but I’d almost gone as a proxy to accompany her because her husband wasn’t allowed with her. She had been building up to the surgery for five years. And she needed somebody to go with her.* [Clinical psychologist, CP Focus Group participant].

Our participants described the interpersonal relationships between the patient (and their family) and the HCP as central to the ability of a HCP (or clinical team) to ‘care’ for their patients:

*…so I work in rehab, some of our patients are here for a long period of time in their rehab capacity receiving lots of different treatments. And a big part of that is the families receiving the support and, and not only the patient …but the family being educated and told how to support their child with that rehabilitation journey. …So we teach both parents, or grandparents or sisters and brothers, whoever is that primary carer for that child. And so that support network is shared. And then as soon as it’s possible [the child goes] into the social spaces of the hospital, they go outside the hospital so they can meet further extended family. If they’re safe, they can go home. And so the child [has] the kind of cognitive, emotional social connection with their community, with their family…And at the moment, we have a lockdown on a single parent being able to visit that child at one particular time, and only two parents being able to share that role.…So parents or caregivers or the extended family are passing ships in the night, they have no chance to have time together in that bed space on that ward to process where they’ve been. And then we can’t send the child home until they’re ready for discharge. And they’re not allowed in communal spaces because of the separate bubbles of the wards…there is such a significant detriment to the rehabilitation process and their ability to grieve and go through the cycle of understanding the traumatic event that they've experienced… actually, that is having a massive impact.* [Paediatric physiotherapist, HCP focus group participant].

Common to the experiences of HCP participants was the importance to healthcare delivery of the (often tangible) interpersonal, relational interaction within which healthcare is offered. This was considered by our participants to be an essential component of patient-centred maternity and paediatric services. Although IPC measures protected HCPs and patients from Covid-19, they were experienced as barriers to the relational experience of care and, in some cases, described as negatively affecting a patient’s outcome:

*So yeah, we had the only one parent, which was really difficult, particularly while he was in intensive care, because there were times when we didn’t think he was going to make it. And then both of his parents got symptoms. So they both had to isolate and then they couldn’t come at all. And then throughout his whole hospital stay, we were limited by only one parent being allowed to be in with him. At the time where it’s normally with such a significant burn, we'd be involving the whole family. This child was 20 months old. During the whole period of his rehabilitation his siblings were not allowed to see him. And we would normally do a lot of work for such a significant burn with like siblings, grandparents together, even simple things like going outside, we couldn’t do. It just impacted on everything we did for him really. [H]e didn’t just have a burn, he ended up having quite significant life changing injuries. And normally, we do a lot of rehabilitation in hospital and then for going home, which we weren't able to do. So they actually like went home with a quite disabled child.* [Paediatric physiotherapist, burns specialist, HCP FG participant].

Reflecting on the efforts that had been made to prioritise care and support to the families of paediatric patients and maternity services users, HCPs described feeling disappointed that these changes were so easily swept away:

*As a neonatal community, we’ve spent so many years trying to move away from being very medical and paternalistic to enabling families to be very much involved as part of a team. And we spent so many years trying to pour our energies into that. And that was literally taken away overnight. All of that work was then undone in March [2020], because, suddenly, the fathers of the children or the partners of the women were banned from the hospital. It’s completely against our ethos*…*we’ve just spent such a long time trying to ensure that parents are seen as equal partners and not visitors that that part of our team and…that the family and the context of the family is incredibly important when you’re delivering care to a sick newborn baby or even a healthy newborn baby so and we spent so long doing that and then now that’s just literally all just got pulled away overnight, and it was it feels really really sad and demoralising.* [Interview, Consultant Neonatologist].

The same concerns were reflected in the public focus groups. Participants described the detrimental impact of IPC measures on the caring relationships that they regarded as particularly important in paediatric and maternity services, such as the involvement of birth partners in ante-natal care and during labour, and the involvement of parents in their child’s hospital care:

*During my pregnancy, I suffered from really bad perinatal anxiety, had secondary PTSD and birth trauma. My partner wasn’t allowed to be there with me. For any of my appointments. I had a bad experience with a consultant. You know, put the fear of God up me. And to do all of that, alone, I gave birth in a mask. So my son saw me—that was his first, you know, image of me.* [Participant, public focus group].

### Where pandemic and everyday ethics collide: specifying the theories and principles engaged

Symbiotic empirical ethics is underpinned by the Aristotelian view that ethical principles need to be adapted both to fit, and to be rendered meaningful in, a particular situation [10]. Thus, while they might be formulated in the abstract, ethical principles are made meaningful in a particular context where they are ‘translated’ into workable guides to everyday actions [34]. For this reason, empirical findings can be used to assess the extent to which ethical theories or principles ‘fit’ in a particular practical context, and suggest where (and how) a particular specification might render them more meaningful. Here we are concerned with the ethical principles that underpin clinical ethics. These, in summary, impose an ethical obligation on an HCP to benefit their patient, to avoid (or minimise) harm, and to respect the patient’s values and preferences [35]. Findings from the Reset project demonstrated a collision in non-Covid clinical practice between ‘everyday’ clinical ethics and the features of ‘pandemic’, public health orientated, ethics, where the focus was the prevention and control of the spread of the coronavirus, often at the expense of the values and preferences of families and hospital communities.

This tension between the contrasting ethical principles and orientations of ‘everyday’ care and ‘pandemic’ care characterised and situated the experiences of our participants. As the first wave of the pandemic hit the UK, the national decision-making structures that were imposed (of necessity) took a one-size-fits-all approach. In the changed working practices which, underpinned by a public health approach, aimed to protect the hospital community and the wider population from the spread of the virus, HCPs came up against practical barriers to being able to provide the type of patient-centred care that is required by clinical ethics:

*There’s just no perfect PPE. So you’ve got masks that you wear, but they’re disposable and they run out and they give you pressure areas on your face and they don’t fit everybody. The hoods [have] these air packs with them [to] recycle the air in the hood. Can’t hear a thing. Can’t hear a thing with them. Can’t put a stethoscope in. Ridiculous! So when I’m taking the blood out of someone’s body, I’m running it through a pump, and I’m putting back into them [there’s] a very real risk of entraining air with that. And I did not realise how much I’m always listening for that—I’m listening for bubbles all the time. And I didn’t realise until I put that hood on that I can't hear anything.* [Nurse, paediatric intensive care, interview participant.]

In the strict imposition of pandemic measures, particularly in the early months, there was often no room for professional discretion or ‘softening’ of the rules to accommodate individual patients and their families. The result was a level of care that, for many HCPs, not only fell short of their professional ethical obligations, but also went against their personal moral codes and impacted on how they felt as a midwife, nurse or doctor. This presented significant ethical challenges for them, which became characteristic of the ‘resetting’ of healthcare services generally, and of maternity and children’s services in particular:

*So when you’re COVID, positive, you’re not allowed to be on the unit. And so, for instance, I probably spent around an hour and a half trying to sort out a sick child, but mainly trying to sort out the dad who was refusing to go home and saying he was going to commit suicide, and then he Facetimes the mum who’s crying. And that seems to fall to the clinical staff to sort out. I spoke to middle management about that one, and they [said] well make sure he doesn't harm [himself]. I’m not a psychologist, how do you want me to assess whether what he’s saying is true or not? It’s probably not. But that’s just—I don’t know. And if he does go and harm [himself], is that on me then? So that's really tricky. And also I can see his point of view, he’s looked after his complex child for five years. Why would you now want to entrust it to strangers to look after, when they’re at their most ill? So I can see his point of view. So that’s quite emotionally draining, having to deal with that.* [Advanced nurse practitioner, paediatric intensive care. Interview participant.]

However, even where discretion was permitted, some HCPs felt unable to take the initiative, having adapted to the strict rules that had been in place for some time, and in light of the potential implications of infection for both patients and colleagues. This was particularly noted in teams where colleagues had died of the virus, or where staff had seen and supported patients and their families who had become infected and been required to follow the strictest IPC measures:

*And we did amend our visiting policies in line with other trusts to, we call it ‘compassionate visiting’, but actually, it is an individual decision, you know, so you have your very young ladies, you have your maybe learning…needs ladies, you had, perhaps very anxious women that have previously been traumatised, and couldn’t face even being admitted without somebody there. It was those kind of marginalised women for one reason or another, that we were trying to strike the balance. But what was interesting was that staff didn’t enjoy that at all. Staff wanted to be told you do this, and then that’s it. So they would stick amazingly to its one partner, nobody else. And even though we launched a policy, we discussed it, we co-produced it, staff would come to the door and say, you know, I've got this woman, and this is the situation, can she have her mother with her? And they wouldn’t do it without me saying so. And I said it’s your call, you know, make it. I will support it. But that took a very long time. And I think that’s perhaps to do with the level of anxiety that people felt and didn’t want to do the wrong thing.* [Head of Midwifery. Interview participant.]

### Using ethical theory as a tool of analysis

In this element of symbiotic empirical ethics, moral concepts are understood as a body of knowledge that can be used as a lens through which to examine the interplay between practice, social and organisational roles, and prescriptive principles [10]. Ethical theories and principles can be used as tools for elucidating and analysing the data, just as, for example, sociologists use theories of social interaction to approach their data. We used ethical theories to discern the areas of disagreement, to clarify terms that are used, and to reveal ambiguities.

A clinical psychologist participating in the CP focus group told us that, during the pandemic, psychological support had increasingly been sought by HCPs due to the impact of working practices on their wellbeing. For the clinical psychologists, this was a notable change to their pre-pandemic practice, where their support would generally be sought by HCPs negotiating difficult circumstances or decisions with patients and families. Changes in working practices led to HCPs finding themselves in situations where they were unable to act to support families in ways that they felt were morally necessary. To compensate for this, they described having to step outside what might usually have been the boundaries of their role and take on additional emotional burdens:

*It was really heartbreaking at times, because the mum especially had a really difficult time. And she did seek psychological support, but she couldn’t have the normal support from her from her partner and her family that you would get at these kind of situations. And it then relied on us to be part of that support, which was very hard on us emotionally as well. I mean the situation in itself was quite hard emotionally. But to have that added extra, it made it really challenging at times.* [Paediatric physiotherapist, burns specialist, clinician FG participant].

The initial thematic analysis of our interview data suggested that the challenges identified by interview participants were related to the ethical importance of *relational* aspects of care. In many cases, the difficulties described were linked to the national mandating of IPC measures, particularly social distancing, visitor restrictions and PPE. These had differing (and sometimes cumulative) effects as the pandemic progressed:

*Safety takes precedence, but then there’s the human side to it. And we know we’ve done harm. And the harm isn’t perhaps that visible, because we’re dealing with young, quite resilient women that have babies, so they're healthy to begin with, and hopefully healthy throughout. But you know, excluding partners or significant others at scans or some of them missing birth, you know, you don’t get that back. And, you know, I think women who’ve had a difficult time or had to be in the hospital for a prolonged period of time, it felt quite isolated. Many women would have shielded for weeks prior up to the birth, and very used to that environment and then suddenly they’re by themselves. And so it’s about weighing up what is safe and what is human, and where do we strike it?* [Head of Midwifery. Interview participant.]

*And…a couple of patients that I’ve looked after that they never saw their child together. The only time they saw their child together in six weeks, was to receive bad news. And then we withdrew treatment. And that’s the only time that they ever saw him together. So I find that’s going to be difficult for people’s grief. And they won’t have any common stories. So—sorry it upsets me a bit actually—So their stories together when they sit at home and think about him and talk about him, [t]hey haven’t got that together. And I think that’s a really difficult casualty of the pandemic. I think that's very hard.* [Nurse, paediatric intensive care].

It seems, then, that the prioritisation of community protection at the expense of the family-centred relationships within which care is offered and experienced in paediatric and maternity services, was often the locus of the harms described. The difficulties discussed were not with the IPC measures per se, as these were acknowledged to be crucially important, but in the typical deferral to the protection of *physical* health over a broader attention to the *emotional* impact of HCPs’ inability to adhere to the ethical norms which guide their professional (and personal) clinical practice. The removal of professional discretion from HCPs in determining how best to put each patient at the centre of decisions about their care, often as part of a broader engagement with their family or caring network, was a key factor. Being required to act contrary to these relational interests in offering care and their own professional autonomy created, for many of our participants, a disjunct between their daily professional practice and both their professional and personal moral codes.

### Relationships recalibrated: theory building from the experiences in our data

The basis of the symbiotic empirical ethics method is that the relationship between theory and practice is not linear. Frith describes how theory can be used to approach the data and how it arises *from* the data, so that the data inform a modification or extension of the theory. In this way, theory interprets data and data interpret theory – and the two processes can occur in the same study. Thus, in symbiotic empirical ethics, theory can be used both for its explanatory power *and* to make normative suggestions. Where ethical dilemmas arise in practice, the practical context can inform the development of ethical theory, the aim being to construct ethical theories that are responsive to the problems experienced in practice. Theory is thus based in (and responsive to) experience, and empirical data are a key aspect of the reformulation of ethical theories. Using this symbiotic relationship between data and theory, and having identified the ethical significance of relationships in clinical practice, we will suggest that, by explicitly attending to the importance of relationships, clinical ethics might both support clinician and patient well-being *and* assist decision-making in the healthcare context.

Our data showed that relationships, as the context for caring, were significantly impacted by the resetting of healthcare services. The importance of public health IPC measures during the pandemic resulted in a ‘frame shifting’ in the NHS from an individual patient to a population-based, public-health perspective [36]. This does not mean that respect for individual patients necessarily became less prominent, rather that the ‘frame shifting’ changed the context for *interpreting* the requirements imposed on HCPs by professional ethics, particularly the expectation in the current GMC guidance that doctors make the care of each patient their first concern.^8^ HCPs described how they could not look after patients in the way they wanted to. IPC measures meant that ‘good enough’ care, provided at a distance and from behind the barrier of PPE, was often all that was available to patients and their families, sometimes in particularly emotionally and ethically challenging situations. The tension of this ‘collision’ between pandemic ethics and the usual, everyday norms of clinical ethics was felt in the networks of relationships connecting patients, the public and members of NHS staff.

Relational theorists suggest that it is in these networks of relationships that our identities are constituted [5]. Notions central to the idea that human beings are separate, bounded individuals are thus re-considered and re-imagined by reference to how we see ourselves in relation to other(s), with face-to-face encounters being accorded a particular significance by some philosophers [28]. In the organisational context, Raul Lejano has suggested that a logic of relationality, where relationality describes the patterns and workings of relationships, better reflects how people work together in practice [37]. He contrasts this with a logic underpinned by *rationality*, which he describes as prescription, guided by reason and knowledge, for pursuing desired ends, where the aim is to maximise the degree to which a decision conforms to a specified criterion. Arguing that a rational, output-driven approach to rules and policies fails to attend to the way people work within relationships, Lejano points to the ‘gap’ that often develops in practice between the design of the rules, or policies, and the way that they work (or are interpreted) by people whose work they are intended to direct.

The application of Lejano’s relational logic to healthcare practice would understand HCPs, patients and their families not just as rule-setting and rule-following beings, but as *relational agents*. In the ‘everyday’ context, interpersonal interactions between colleagues, patients and families would determine how to fit the rules around a particular clinical set of circumstances to best meet a particular patient’s needs. In the emergency (or pandemic) context, the moral significance of relational engagement might not always outweigh other concerns, but an explicit recognition of the implications of ignoring human relationships might advocate for a more dynamic approach to (say) IPC measures when a new disease becomes better understood. Relational logic thereby prioritises the sequences of actions and reactions that express and reinforce relationships – between members of the healthcare team, the team and the patient, the team and the patient’s family, and between the family members themselves. The aim is understanding and consensus within the spirit, if not always the letter, of hospital policy. Viewed in this way, the rules are (to an extent) dynamic and create an institutional approach which is *negotiated* to accommodate relational interactions and priorities [37]. In the healthcare context, these negotiations might involve HCPs and patients, colleagues, or managers and staff depending on the policy or process in question. Such a negotiation might even comprise a ‘bottom up’ process, where grass roots experience informs organisational change.

An approach underpinned by a relational logic understands people *within* their networks (patients, HCPs and families) as tending outwards, taking responsibility for what their actions mean in the life of another [38], being constituted within and by their *relationships*, rather than existing as autonomous individuals in the Cartesian sense [39]. Gómez-Vírseda and Usanos, against the backdrop of the pandemic, have developed a multi-layered account of relationality in the context of bioethics [14], and our findings provide empirical evidence of the importance of these theoretical ideas in healthcare practice. Key for members of the public and HCPs were emotional engagement and what we might characterise as the meeting of relational needs, such as the sharing of significant life events, supporting others in challenging circumstances, and making decisions together with others. The caring relationships *between* HCPs and their patients, and the families of their patients, can then, be understood as working to (co-)constitute their identities as *people-in-relationships*, offering care and support and, in so doing, shaping both professional and personal relational selves. Our qualitative data have re-emphasised the significance of those relationships by exposing the harms that have resulted from damaging them:

*We begged the trust to be able to let this woman in, she had two negative COVID tests [but] the trust wouldn’t budge with letting her into the unit because she had to quarantine for two weeks. And the baby started deteriorating on day five, and gradually got worse. I asked the trust again to let her in. And they said no, because the baby wasn’t for end of life care. So the only time that she’d be allowed in was if the baby died. And the baby did die on day seven. And that’s the only time she saw her baby. You get annoyed with [management] and angry with them. And you think, you know, you just know you're following a government guideline*. [Neonatologist, Interview participant].

The strict public health measures which were intended (understandably) to protect hospital communities and the ability of the healthcare system to continue to function, restricted (and in some cases removed) HCPs’ usual ability to negotiate or interpret policies and guidelines *with* their patients (and families) to fit the particular therapeutic context. Where HCPs had some discretion, there were worries about the consequences on patients’ relationships if they got the decision wrong and someone contracted Covid-19 or if other patients challenged a perceived unfairness. Attending in our analysis to the central importance of relationships in our data, we can theorise that the challenges described and experienced by our participants were linked to the generally non-negotiable nature of the IPC measures imposed during the pandemic.

We can make sense of our findings through the lens of the philosophical picture painted by Gómez-Vírseda and Usanos, in combination with Lejano’s logic of relationality. Clinical ethics is underpinned by the perception of the patient as ‘similar to that of the Cartesian cogito: a rational, self-interested, transparent entity for whom personal wishes and volition are fully conscious’ [14]. The role of the HCP, in this analysis, is reduced to the provision of medical options to inform the patient’s choice [40]. We thus posit that, generally speaking, hospital policy and organisational decision-making sit within an explicit logic of rationality, in terms of the formulation of policy and guidance. However, when these policies and guidelines come to be interpreted and applied in practice, HCPs engage a logic of relationality.^9^ Relationality might be prioritised to different extents in different settings and specialties. While the rational aim of policy to support government targets, management objectives or wider regulatory requirements is explicit, the relational interpretation is largely *implicit*, part of the ‘humanness’ of everyday practices of care. The GMC guidance, as we have seen, requires doctors to make their patient their first concern and to treat patients as individuals [12]; there is no explicit attention to the ‘patient-in-relationships’, despite the fact that in pre-Covid practice the patient’s family was considered part of the team in maternity and paediatric services. However, pre-Covid practice notwithstanding, in this acute emergency, IPC measures were often not negotiable; the caring relationships *had* to give way:

*But it’s also really stressful with our parents who are very experienced, very involved in their children’s care, for them to be excluded, because they really are part of our team. They are part of the treatment team for the child. Rather than just a supportive parent. Actually, it’s really hard for staff to turn them away, because you know what it is that they’re doing, and that they can. So I’d say probably that has been the greatest challenge for us*. [Paediatric intensive care consultant, Interview participant].

## Discussion and conclusions – re-imagining clinical ethics?

We have argued that foregrounding the importance of relationships for the wellbeing of people across a hospital community will better promote the ethically important multi-directional expression of caring between HCPs, patients, and their families. We do not claim that relationships are currently unrecognised in healthcare decision-making—this is clearly not the case. Rather, our argument is that when considering what policies and procedures to implement there should be an *explicit* consideration of the potential impact on *relationships*, as well as *rational* outcomes (IPC measures, for example). It is equally important that this approach should be a feature of all healthcare decision-making, not just when services are stretched, or in pandemics and other emergencies. We offer two suggestions for making progress towards such a relational approach. First, that there is a change of emphasis in clinical ethics to explicitly acknowledge the importance of the relationships (including between healthcare team members) within which the patient is held [14]. Second, that organisational decision-making should account for the moral significance afforded to caring relationships by HCPs, and the role such relationships can play in the negotiation of ethical challenges.

To the first suggestion, the pandemic context magnified the difficulties for HCPs in negotiating the tensions between professional obligations to individual patients and obligations to the wider community [41]. By recognising the importance of the relational, the benefits to patients of the support of their families would become part of HCPs’ professional concern. In practice, of course, HCPs already attend to concerns more widely drawn than the interests of an individual patient [17], and this supports our contention for the importance of the relational to be made explicit. So, for instance, guidance for aspiring neonatologists states that, in this specialty ‘the level of family integrated care is unparalleled, and any practised doctor must understand the holistic nature of care required to build a good rapport with the families’ [42]. Similarly, guidance promulgated by the Royal College of Midwives references a midwife’s role as the provision of ‘support [for] women and their families throughout the childbearing process to help them adjust to their parental role’ [43]. Furthermore, the NHS is built around, and underpinned by, relational values and concerns [44]. The importance of attending specifically to the ‘patient-in-relationships’ rather than the patient as an individual is, thus, made clear in professional guidance for neonatologists and midwives working within the NHS, and this should be recognised in clinical ethics too, as Gómez-Vírseda and Usanos have persuasively argued [14].

The second suggestion extends the first. Recognising the importance of relational networks in reimagining the parameters of clinical ethics requires similar attentiveness to their importance in organisational decision-making more broadly. Our data show that good relationships with colleagues and patients are fundamental to HCPs’ wellbeing, particularly when ethically challenging decisions are required. It follows, then, that hospitals (and other healthcare settings) should consider the possible benefits of a policy and decision-making approach grounded in (or at least explicitly attentive to) a logic of relationality. An approach to decision-making underpinned by a specific intention to value a richer mix of human experiences in the conception of the patient, and a wider range of what are considered relevant outcomes, is thus required. There may be something to be learned here from the relational and social approach taken by hospices caring for people approaching the end of life. In that context, an attention to a patient’s ‘total pain’, encompassing not only physical but also the emotional, social and spiritual aspects of each patient’s experience, has ‘reframed the relationship between medical professionals and dying patients’ [45]. Both the current and the previous versions of the GMC guidance require HCPs, in assessing a patient’s condition and history, to take account of psychological, spiritual, social, economic and cultural factors, as well as the patient’s views, needs and values [46]. It would not seem too significant a shift to accord these factors, including the patients’ significant supportive relationships, a greater role in organisational level decision-making.

The Reset project data indicate that, for patients and for HCPs, interpersonal relationships are fundamentally important to *care*, and that healthcare without that relational engagement becomes *functional treatment*, which is something different [47]. Lejano similarly emphasises the importance of relational engagement, contending that interpersonal relationships and everyday transactions are the mechanisms used to work policy into practice, so that policy becomes ‘the workings of relationships’ [37]. This is equally true for the practical expression of the requirements of clinical ethics, where an increased emphasis on the ‘humanness’ recognised by and central to *Igwebuike* as a means of stress testing policies and working practices might help improve decision-making and staff and patient wellbeing too. We contend that a shift in the emphasis of clinical ethics to encompass the ‘*patient-in-relationships*’ as the focus of a HCP’s concern would therefore represent a readily defensible normative suggestion.

### Supplementary Information

Supplementary Material 1. 

## Data Availability

All anonymised participant data that support the findings reported in this study are available on reasonable request to the corresponding author.

## Footnotes

1 At the beginning of the pandemic, many non-Covid-19 healthcare services in the UK were suspended. In April 2020, the Department of Health and Social Care, in England, mandated that non-Covid services should resume alongside the continuing pandemic response. See Stevens S, Pritchard A. Second phase of NHS response to COVID-19, 2020. Available: https://www.england.nhs.uk/coronavirus/wp-content/uploads/sites/52/2020/04/second-phase-of-nhs-response-to-covid-19-letter-to-chief-execs-29-april-2020.pdf

2 The NHS Reset Ethics research was funded by the UKRI AHRC rapid Covid call (AH/V00820X/1) and ran from July 2020 – September 2021. The project was conducted by a multi-disciplinary team including the authors (PI Dr Lucy Frith) with Professors Heather Draper and Sara Fovargue, Dr Anna Chiumento and Dr Paul Baines (previously of The University of Warwick.

3 See Institute for Government's timeline of UK coronavirus lockdowns, March 2020 to March 2021: https://www.instituteforgovernment.org.uk/sites/default/files/timeline-lockdown-web.pdf

4 A new version of GMC guidance came into force in January 2024. The effects of the changes in the revised Guidance are considered below.

5 As to which, see further below.

6 Note that a detailed description of our empirical methods is provided in Chiumento et al. [4]. The summary description here is intended to underpin the discussion of the symbiotic empirical ethics methodology that is the focus of this article.

7 Six NHS trusts were recruited to the study, but only five took part. At one trust, pressure of work meant that we were unable to recruit any senior managers or HCPs to participate in interviews or focus groups.

8 We note that, in practice, this is (and has for some time been) largely impossible for HCPs to achieve, even in ‘usual’ times, see, for example, Sokol, D. (2011). Make the care of your patient your first concern. *The BMJ*. 342. This, in our view, increases the urgency of reviewing a more appropriate ethical standard for healthcare practice.

9 Lejano notes that relational processes function along with rational/purposive rule systems in complementary fashion, and that we should expect to find the relational to be operative everywhere, even in programmes that conform strictly to set rules and formal guidelines: Lejano, R. (2021). Relationality: an alternative framework for analysing policy. Journal of Pub Policy; 2021:41:22: 360–383.
