# Supplementary material for: Using symbiotic empirical ethics to explore the significance of relationships to clinical ethics: findings from the Reset Ethics research project
Source: BMC Med Ethics. 2024 May 28;25:66. doi: 10.1186/s12910-024-01053-9 (PMC11131179; doi:10.1186/s12910-024-01053-9)
Supplement: Supplementary file 1 — Supplementary Material 1. [file 12910_2024_1053_MOESM1_ESM.docx]

**ADDITIONAL FILE 1: Interview and focus group topic guides**

**PART A: Interview topic guides**

**Topic guide: senior manager interviews**

**Research ethics application number: [ ]**

**IRAS ID: [ ]**

**Title of the research project:** When pandemic and everyday ethics collide: supporting ethical decision-making in maternity care and paediatrics during the Covid-19 pandemic – NHS Reset Ethics Project

**Broad approach**

An important focus of these interviews is the decision-making *processes* incorporating an interest in

- *Approaches* to decision-making
- *Decisions* made
- The *justifications* for those decisions

However – the *ethical values* guiding/underpinning/’marbling through’ these processes (implicit and explicit) are what we are looking to explore and tease out.

To strip away the layers of the onion (to the extent we can in the time we will have), our approach will be to ask a number of broad general questions followed by gentle probes, as appropriate to the response of each participant - ie not all probes for all participants. The semi-structured approach means we will be guided by the participant’s responses, but have the probes, and the issues they are designed to elicit, in mind as the conversation progresses. The order in which the areas touched on in the topic guide are covered will vary according to how issues are introduced/addressed by participants.

We will approach the discussion mindful of the empirical ethics requirement that participants be encouraged to provide some reasons or reasoning for the views they hold. To elicit this data, in addition to the gentle probing of views mentioned above, we may also need to consider more active facilitation. This can take the form of e.g. suggesting (in an unheated and gentle way) counter-arguments to participants’ positions, the aim being to encourage participants to explore their own positions more fully.

**Introductory comments**

Thank you very much for agreeing to talk to me/us today. Just to remind you, this interview will be recorded and I will tell you when the recording starts. You do not have to answer all of our questions, and if you want to stop at any time, that’s fine Just let me know.

Just to remind you, our project is focussed on the ethical aspects of decision-making during the ‘reset’ phase of the current pandemic. That is to say, the point after the initial acute phase of the pandemic, when Trusts were asked to re-establish the services that had been suspended to allow for a focus on treating patients with Covid-19 during the first wave. We are also taking into account the difficulties that might arise in maintaining services during any subsequent spikes and waves.

Our particular focus is on two areas of non-covid-19 healthcare: maternity services and paediatric surgery.

We/I would like to discuss your experiences of leading decision-making in the Trust during the recovery phase after the first wave of the Covid-19 pandemic. We/I would like to explore your experiences of the challenges you have faced during this difficult period, focussing in particular on how you ensured that decisions were acceptable and justifiable.

***Please share your examples of situations and challenges / difficulties you faced, whilst being mindful of confidentiality, as this really helps us to understand your experiences in context.***

Do you have any questions about the project or this interview that I can answer before we start?

OK, I am going to turn on the recording now.

TURN ON RECORDING AND CHECK WORKING

Ok now I am recording.

WARM UP QUESTION (5 mins)

Please describe your current role(s) and responsibilities and a brief summary of your previous role(s) within the trust

MAIN QUESTIONS (4 x 10 minutes each)

1. HOW DID YOUR TRUST APPROACH RESTARTING THOSE SERVICES THAT WERE SUSPENDED?

**Probes**:

- What informed decision-making as the Trust planned to restart services towards the end of the first acute phase of C19 (i.e. May to June 2020)?

**Explore**: guidelines / policies / budget / human resource constraints etc that informed decision-making.

- What (if any) guidelines / policies did the trust draw upon? How did you use them? Were they useful? Could we have a copy (I will remind you about this later)?

**Explore:** underpinnings of guidelines/policies eg safety / triage / etc

- What existing or new structures were involved in making these decisions? (e.g. committees, patient involvement)

**Explore:** membership of any committees / how patient involvement facilitated – user groups / charities etc

- How did you reach agreement about what the priorities were for restarting?

1. WHICH DECISIONS ABOUT HOW BEST TO RESET SERVICES WERE DIFFICULT OR UNCOMFORTABLE TO MAKE?

**Probes:**

- Attempt to clarify who felt uncomfortable and why/what seemed to be the source of discomfort

**Explore:** what did you worry about most? Why did you worry about that most?

- Attempt to draw out what the particular ethical tension, if there was one, that created this discomfort – express it as an ethical tension so that participant aware that this is ‘ethics’
- How were these issues considered and resolved? Can you talk me/us through a particular example?
- If some issues could not be resolved, why do you think that was?

**Explore:** whether / how have you managed to come to terms with that worry?

- Did you try and reach consensus, vote? How did you resolve disagreement
- If all challenges were resolved satisfactorily, what do you think contributed most to ensuring this?

1. WHAT (ETHICS) ADVICE OR SUPPORT COULD YOU ACCESS TO HELP YOU DEAL WITH THESE UNCOMFORTABLE DECISIONS?

**Probes:**

- Your Trust has a clinical ethics committee, how useful was the CEC at this time?
- How did you access the CEC’s support?
- What kind of cases/policies did you seek a view on from the CEC?
- What sort of documentation was used when accessing this support? **Could we have copies of this documentation (blank forms are fine)? [I’ll remind you about this at the end]**
- If you disagreed with that group’s recommendations or advice, how did you proceed?
- If you did not have access to an ethics support group, what would have been useful and why?

1. SOMETHING WE ARE INTERESTED IN EXPLORING IN THIS PROJECT IS HOW THINGS LIKE RISKS OF HARM (FOR STAFF AND SERVICE USERS), SERVICE COSTS, AND MAINTAINING C19 CARE ALONGSIDE ‘NORMAL’ SERVICES ARE BEING BALANCED. WE MIGHT CALL THIS THE “NEW NORMAL”. HOW DO YOU SEE “THE NEW NORMAL” CHANGING YOUR TRUST’S DECISION-MAKING?

TO WHAT EXTENT DO YOU THINK THAT THE VALUES THAT GUIDED HOW SERVICES WERE PROVIDED IN THE TRUST BEFORE THE PANDEMIC HAVE BEEN DISRUPTED BY THE PANDEMIC?

**Probes:**

- It has been suggested that at the height of the first wave, there was a greater emphasis on the public health dimension of ethical decision-making. This focused on ensuring the best outcome for as many people as possible. This was seen as a tendency to a more utilitarian way of thinking about ethical decision-making. To what extent do you think it has been possible to temper this emphasis on protecting the interests of the population above all else with protecting the interests of individual patients, including respecting their autonomy as the pandemic has progressed and suspended services have been reintroduced?
- What do you feel has been ‘thrown under the bus’ in the process and what concerns does that raise for you?
- Are there any aspects of the ‘new normal’ that are preferable to practice before the pandemic?

**Explore:** eg telemedicine / visiting changes / reduced numbers of people in the hospital

- If so, could you describe how and why?
- To what extent have you been able to evaluate these changes with frontline staff / service users?

**Explore:** evaluating acceptability, effectiveness, uptake of new practice across departments or integration into SOPs etc: are these evaluation initiatives seen as helpful or burdensome?

- To what extent has decision-making shifted from a more individual patient focus to a public-health oriented approach?

WRAP-UP QUESTIONS (10 mins)

1. Thinking about support or training for making service reset decisions or responding to ethical issues, what would you find useful?
2. Is there anything we have touched on that you would like to return to or discuss in more detail?
3. Is there anything you would like to discuss that you feel is important but hasn’t been touched on?
4. And finally, you mentioned that you would be able to send me copies of some of the documents your Trust used to support reset decision-making. Can I email you to confirm the copy documents we are interested in? Are any of them available through your website?

OK good: I am going to turn off the recording now

TURN OFF

I am no longer recording.

Thank you – etc.

**Interview topic guide: healthcare professionals**

**Research ethics application number: [ ]**

**IRAS ID: [ ]**

**Title of the research project:** When pandemic and everyday ethics collide: supporting ethical decision-making in maternity care and paediatrics during the Covid-19 pandemic – NHS Reset Ethics Project

**Broad approach**

The key focus in these interviews is the way(s) the working practices of healthcare professionals have changed to accommodate Covid-19/the pandemic since the May/June restarting/reset of services. The aim is to explore how they feel about these changes and any ethical challenges/difficulties that they have experienced/encountered as a result.

The questions therefore are trying to elicit the lived experiences of staff applying changed policies to their clinical practice. This includes *pandemic-related policies* that are being applied into the reset period (eg visitor/accompanying support person policies) and *reset-related policies*.

The *ethical challenges/difficulties* encountered by health care professionals as they live with the changed ways of working these policies have effected to daily routines and decision-making processes (implicit and explicit) are what we are looking to explore and tease out.

To strip away the layers of the onion (to the extent we can in the time we will have), our approach will be to ask a number of broad general questions followed by gentle probes, as appropriate to the response of each participant - ie not all probes for all participants. The semi-structured approach means we will be guided by the participant’s responses, but have the probes, and the issues they are designed to elicit, in mind as the conversation progresses. The order in which the areas touched on in the topic guide are covered will vary according to how issues are introduced/addressed by participants.

We will approach the discussion mindful of the empirical ethics requirement that participants be encouraged to provide some reasons or reasoning for the views they hold. To elicit this data, in addition to the gentle probing of views mentioned above, we may also need to consider more active facilitation. This can take the form of e.g. suggesting (in an unheated and gentle way) counter-arguments to participants’ positions, the aim being to encourage participants to reflect more fully on their own experiences of policy implementation and decision-making in the ‘reset’ phase of the pandemic, particularly where they found decisions/policy implementation uncomfortable or worrying.

**Introductory comments**

Thank you very much for agreeing to talk to me/us today. Just to remind you, this interview will be recorded and I will tell you when the recording starts. You do not have to answer all of our questions, and if you want to stop at any time, that’s fine, just let me know.

Just to remind you, our project is focussed on the ethical aspects of decision-making during the ‘reset’ phase of the current pandemic. So from around May/June 2020, the time when Trusts began re-establishing the services that had been suspended to allow for a focus on treating patients with Covid-19 during the first wave. We are also taking into account the difficulties that might have arisen (or be arising now) in maintaining non-Covid related services during the pressures created by the current second wave of the virus.

Our particular focus is on two areas of non-covid-19 healthcare: maternity services and paediatric surgery.

We/I would like to discuss how your working practices have had to change as a result of covid-related policies being applied in your Trust and affecting your clinical practice, and service delivery to patients and families, from May or June 2020, or from the time when non-covid services resumed. We/I would like to explore your experiences of the challenges you have faced during this period, focussing in particular on any difficult situations you have experienced as a result of the changes made to service delivery and to your working practices, any ethical concerns these might have raised for you and how you dealt with them.

***Please share your examples of situations and challenges / difficulties you faced, whilst being mindful of confidentiality, as this really helps us to understand your experiences in context.***

Do you have any questions about the project or this interview that I can answer before we start?

OK, I am going to turn on the recording now.

TURN ON RECORDING AND CHECK WORKING

Ok now I am recording.

WARM UP QUESTION (5 mins)

Please describe your current role(s) and responsibilities within the Trust [and a brief summary of any changes due to Covid-19 on your job role since March 2020.]

MAIN QUESTIONS (4 x 10 minutes each)

1. HOW HAVE YOUR PRACTICES HAD TO CHANGE TO ACCOMMODATE COVID/ THE PANDEMIC SINCE THE RESTARTING OF NON-COVID SERVICES [IN MAY/JUNE OF THIS YEAR/LAST SUMMER]?

**Probes**:

- How frequently have changes been made to working practices? How have they been communicated? Has there been any training offered?

**Explore:** how that has made the team feel**.**

- What do you know about the policies driving /underpinning these changes?

**Explore**: guidelines / changed ways of working / PPE / telemedicine / home visits (in maternity) /differences in team structure to accommodate staff illness/self-isolation/shielding/ redeployment of you or other members of your team likely to be more at risk (eg BAME colleagues) to other duties.

- What (if any) guidelines / policies did your service/team draw upon? Were they useful? How have revised policies been communicated to you? Was the team able to input into the development of any changed ways of working?

**Explore:** how they understood the reasons for the changes/ the underpinnings of new guidelines/policies eg safety / identifying clinical priorities/balancing urgent care scenarios / fairness etc

- How, in your experience, how have patients/families been responding to new ways of working? Have there been any specific situations you can describe?

**Explore:** whether/how patient feedback to any changes has been received – has it been possible to accommodate patient wishes? If not, how has that been managed? [ANY DOCUMENTS COULD SHARE RE PATIENT FEEDBACK]

- Have new ways of working caused you any personal difficulties? (eg asymptomatic birth partners testing positive means increased risk for HCPs/PPE required for HCPs involved in the birth)
- How have these changes to your working practices made you feel about the care you have been able to provide?
- How have team members been able to support each other in adapting to new ways of working?

1. CAN YOU DESCRIBE WHETHER THERE HAS BEEN ANY IMPACT ON THE VALUES THAT GUIDE SERVICE PROVISION AS A RESULT OF THE C19 PANDEMIC / CHANGES IN PRACTICES?

**Probes**:

- What do you think you are having to take into account alongside individual patient/family needs/interests?
- How do you think things like risks of harm (for staff and service users), access to services, and maintaining c19 care alongside ‘normal’ services are being balanced? Can you describe what you think is being done well/not so well?
- How have you been able to make sure, to your own satisfaction, that your patients understand why things are being done differently?
- How have you been able to make sure that your patient’s/their family’s choices, wishes and feelings have been taken into account? If that *hasn’t* been possible, how have you managed the situation?
- How have you been able to take care of your own, and your colleagues’, safety and well-being?
- What do you feel has been ‘thrown under the bus’ with your changed ways of working and what concerns does that raise for you?

1. HOW HAVE YOUR CHANGED WORKING PRACTICES RESULTED IN SITUATIONS THAT YOU HAVE FOUND DIFFICULT OR UNCOMFORTABLE?

**Probes:**

- Attempt to clarify who felt uncomfortable and why/what seemed to be the source of discomfort

**Explore:** what did you worry about most? Why did you worry about that most?

- Attempt to draw out what the particular ethical tension, if there was one, that created this discomfort – express it as an ethical tension so that participant aware that this is ‘ethics’
- How were these situations considered and resolved? Can you talk me/us through a particular example?
- If some issues/situations could not be resolved, why do you think that was?

**Explore:** whether / how have you managed to come to terms with that worry?

- If all challenges were resolved satisfactorily, what do you think contributed most to ensuring this?
- How have you and your team members been able to support each other where you/they have experienced difficult or uncomfortable situations?

1. WHAT (ETHICS) ADVICE OR SUPPORT CAN YOU ACCESS TO HELP YOU DEAL WITH THESE DIFFICULT SITUATIONS?

**Probes:**

- Your Trust has a clinical ethics committee; how useful is the CEC to you / colleagues at this time?
- How can you access the CEC’s support?
- Can you describe a situation that was referred to the CEC? Or a situation where referral was considered but not made because the situation was resolved some other way?
- If you disagreed with the CEC’s recommendations or advice, how did you proceed?
- If you do not have access to an ethics support group, what would you find useful to support the team in resolving difficult situations and why?
- Would opportunities for ethical reflection/’debrief’ in relation to these difficulties/worries be valuable?
- If it would be valuable to have an opportunity to reflect on/be ‘debriefed’ about the difficult situations you have encountered, how would you see that working most effectively?

WRAP-UP QUESTIONS (10 mins)

1. What kind of ethical support would you value/find useful? [*If this hasn’t come out in the conversation previously*]
2. Is there anything we have touched on that you would like to return to or discuss in more detail?
3. Is there anything you would like to discuss that you feel is important but hasn’t been touched on?

OK good: I am going to turn off the recording now

TURN OFF

I am no longer recording. Thank you – etc.

**PART B: Focus group topic guides**

**Topic guide: Focus group for the public**

**Research ethics application number: [ ]**

**IRAS ID: [ ]**

**Title of the research project:** When pandemic and everyday ethics collide: supporting ethical decision-making in maternity care and paediatrics during the Covid-19 pandemic – NHS Reset Ethics Project

**Introductory comments**

Thank you very much for agreeing to participate in our focus group discussion today. Just to remind you, the discussion will be recorded and I will tell you when the recording starts. We will use this recording to create a transcript of our discussion for us to analyse. This transcript will be anonymised – removing all mention of places or people’s names. You do not have to answer all the questions, and if you want to stop at any time, that’s fine. Also, if you need to leave the group, please let us know. If you would like to speak while someone else is talking, please raise your hand [demonstrate use of icon].

To recap, our project is focussed on the ethical aspects of decision-making during the ‘reset’ phase of the current pandemic. We want to hear your views about how involved you felt with decisions that your local Trusts made, when they were trying to find the best and fairest ways to solve the problems that Covid-19 caused for maternity services and caring for sick children. We are also interested in what you think about how these decisions should be made when people disagree about what to do, and how the users of these services are or could be involved in making these decisions. As part of the discussion, we would welcome specific examples that illustrate the points you want to make to “bring to life” your experiences

[*if necessary – I will also guide those who have not returned their consent forms through the consent process once we have started recording*]

Do you have any questions about the project or this focus group that I can answer before we start?

OK, I am going to turn on the recording now.

TURN ON RECORDING AND CHECK WORKING

We are now recording. We’ll start with a quick round of introductions – please just use your first or given name to introduce yourself and say which is your local NHS Trust.

[any verbal consent recorded here]

**We are seeking your views on public involvement and/or consultation processes for your local services. It’s perhaps useful to point out that NHS trusts have a legal duty to involve the public in policy decisions that is, in essence, unaffected by C19.**

**Q1:** Can I start by asking why you feel that public involvement is/isn’t important for healthcare decision-making?

**Q2:** How engaged are you with local healthcare decision-making at the moment?

(prompts: involved in public advisory groups, specific actions groups such as maternity voices, involved with support groups for specific conditions)

**Q3:** What form does this involvement take?

(prompts: online meetings, being asked to comment on proposals, lobbying for services, completing online or telephone surveys, previous involvement in research)

**Q4:** What was your level of involvement with local decision-making prior to April/May 2020?

**Q5:** What reduced your previous level of involvement (if applicable)?

(prompts: less opportunity to comment, not being asked for opinions, no communication etc)

**Q6:** What do you think were the Trust’s priorities when making decisions about healthcare as restrictions were eased and services were ‘resetting’ towards normal?

**Q7:** What could Trusts do to facilitate your involvement?

(prompts: better publicising of intended changes, invitations to comment, membership of groups to look at specific aspects of NHS provision)

**Q8:** How could your local Trust better prepare you for more involvement with decision-making processes?

(prompts: training on how the NHS works, how funding works, training on how decisions about healthcare are made (could include ethics))

**Q9:** Is there anything that you would like to discuss that you feel is important or hasn’t been touched on?

Thank you very much for participating. I will now stop the recording.

If you would like more information or support with anything that we have covered today, please see the debrief information that we have emailed to you.

The transcript will be produced using an automated service, and we will then check for any identifying information which will be removed. It will then be analysed alongside our interviews with healthcare professionals. If you would like a copy of the final report, please contact [email address] and we will send you a copy when it is available.

**Topic guide: clinical psychologists focus group**

**Research ethics application number: [ ]**

**IRAS ID: [ ]**

**Title of the research project:** When pandemic and everyday ethics collide: supporting ethical decision-making in maternity care and paediatrics during the Covid-19 pandemic – NHS Reset Ethics Project

**Broad approach**

We will approach the discussion mindful of the empirical ethics requirement that participants be encouraged to provide some reasons or reasoning for the views they hold. To elicit this data, in addition to the gentle probing of views, we may also need to consider more active facilitation. This can take the form of e.g. suggesting (in an unheated and gentle way) counter-arguments to participants’ positions, the aim being to encourage participants to reflect more fully on their own experiences of supporting colleagues/families in the ‘reset’ phase of the pandemic, particularly where they found decisions/policy implementation uncomfortable or worrying.

**Introductory comments**

Thank you very much for agreeing to participate in our focus group discussion today. Just to remind you, the discussion will be recorded and I will tell you when the recording starts. We will use this recording to create a transcript of our discussion for us to analyse. This transcript will be anonymised – removing all mention of places or people’s names.

You do not have to answer all the questions, and if you want to stop at any time, that’s fine. Also, if you need to leave the group, please let us know.

If you would like to speak while someone else is talking, please raise your hand [demonstrate use of icon].

To recap, our project is focussed on the ethical aspects of decision-making during the ‘reset’ phase of the current pandemic. By this, we mean the period after the first wave of the pandemic, when the services that had been shut down were restarted, and ‘reset’ to accommodate covid-related changes. So from around May/June 2020, the time when Trusts began re-establishing the services that had been suspended to allow for a focus on treating patients with Covid-19 during the first wave. We are also taking into account the difficulties that might have arisen (or be arising now) in maintaining non-Covid related services during the pressures created by the current second wave of the virus.

Our particular focus is on two areas of non-covid-19 healthcare: maternity services and paediatric surgery.

As part of the discussion, we would welcome specific examples that illustrate the points you want to make to “bring to life” your experiences

Do you have any questions about the project or this focus group that I can answer before we start?

OK, I am going to turn on the recording now.

TURN ON RECORDING AND CHECK WORKING

We are now recording

1. The pandemic has changed how we all work. Some changes have been positive, others less positive but perceived as necessary. The upheaval has created many big and little changes that we have had to absorb, and which may now even seem normal as we become accustomed to accommodating them.

`What has changed, if anything, in the support you have offered during this ‘reset’ phase?

1. Some of our participants have told us that when they feel distress as a result of the ethical issues they have faced at work, one avenue of support for them is psychological services. To what extent has distress as a result of experiencing difficult ethical issues been the reason colleagues have sought your support during the reset period?

Follow up:

- How do colleagues express their discomfort?
- Where new rules have put pressures on colleagues, how do they navigate this?
- How do you know when colleagues’ distress relates to **ethical** decisions or issues they have faced as a result of their changed working practices?

It may be helpful to think back to a recent case you’ve had, and just briefly describe one example.

1. How does the support you provide for staff who are distressed as a result of ethical issues playing out differ to the support you are already offering during these difficult times?

Follow up:

- What tools do you think you have in your toolbox to help you work through ethical dilemmas to support colleagues?
- Are there specific models or techniques that guide your practice in this area? [*explore whether any of these models are more values-driven, and may fit more naturally with the sort of ethical lens we are proposing.  Exploring this seems important for thinking about whether the ethical lens integrates more into certain types of therapeutic approaches than others*.]
- What works well / what could be changed?

1. If it was up to you:
2. what kinds of things would you put in place to support staff working through these sorts of issues?
3. what kinds of things would help **you** support staff work through these sorts of issues?

Thank you very much for participating. I will now stop the recording.

If you would like more information or support with anything that we have covered today, please see the debrief information that we have emailed to you.

The transcript will be produced using an automated service, and we will then check for any identifying information which will be removed. It will then be analysed alongside our interviews with healthcare professionals. If you would like a copy of the final report, please contact [email address] and we will send you a copy when it is available.

**Topic guide: healthcare professional focus group**

**Research ethics application number: []**

**IRAS ID: []**

**Title of the research project:** When pandemic and everyday ethics collide: supporting ethical decision-making in maternity care and paediatrics during the Covid-19 pandemic – NHS Reset Ethics Project

**Broad approach**

We will approach the discussion mindful of the empirical ethics requirement that participants be encouraged to provide some reasons or reasoning for the views they hold. To elicit this data, in addition to the gentle probing of views, we may also need to consider more active facilitation. This can take the form of e.g. suggesting (in an unheated and gentle way) counter-arguments to participants’ positions, the aim being to encourage participants to reflect more fully on their own experiences of policy implementation and decision-making in the ‘reset’ phase of the pandemic, particularly where they found decisions/policy implementation uncomfortable or worrying.

**Introductory comments**

Thank you very much for agreeing to participate in our focus group discussion today. Just to remind you, the discussion will be recorded and I will tell you when the recording starts. We will use this recording to create a transcript of our discussion for us to analyse. This transcript will be anonymised – removing all mention of places or people’s names.

You do not have to answer all the questions, and if you want to stop at any time, that’s fine. Also, if you need to leave the group, please let us know.

If you would like to speak while someone else is talking, please raise your hand [demonstrate use of icon].

To recap, our project is focussed on the ethical aspects of decision-making during the ‘reset’ phase of the current pandemic. By this, we mean the period after the first wave of the pandemic, when the services that had been shut down were restarted, and ‘reset’ to accommodate covid-related changes. So from around May/June 2020, the time when Trusts began re-establishing the services that had been suspended to allow for a focus on treating patients with Covid-19 during the first wave. We are also taking into account the difficulties that might have arisen (or be arising now) in maintaining non-Covid related services during the pressures created by the current second wave of the virus.

Our particular focus is on two areas of non-covid-19 healthcare: maternity services and paediatric surgery.

As part of the discussion, we would welcome specific examples that illustrate the points you want to make to “bring to life” your experiences

Do you have any questions about the project or this focus group that I can answer before we start?

OK, I am going to turn on the recording now.

TURN ON RECORDING AND CHECK WORKING

We are now recording

1. The pandemic has changed how we all work. Some changes have been positive - maybe telemedicine - others less positive but perceived as necessary – I have mentioned visiting restrictions as an example. But the upheaval has created many big and little changes that we have had to absorb, and which may now even seem normal as we become accustomed to accommodating them – eg wearing masks in the supermarket.

**Are there any changes to your work practices you think some staff would feel uncomfortable about becoming permanent? If so, why do you think that is?**

- How do members of your team express their discomfort?
- To what extent do you feel that their voices are heard?
- What kinds of things might people do to help them feel more comfortable in these situations
- When might you feel that it is really important to make exceptions to these ‘new normals’?
  - To what extent are you able to make exceptions
  - How is this done

**Which, if any, changes in how you care for patients that you would not want to see normalised?**

2. How do you/your team know when you are making an *ethical decision / ‘doing ethics*’?

It may be helpful to think back to the practices we have just been discussing, OR most recent shift you’ve done, and just briefly describe one such occasion from that shift.

(If someone can’t think of anything from their most recent shift, just open out to a shift in the last month or so)

Follow up

- how was the problem/issue resolved / how was the decision made
- what happened next/ who decided in the end
- how could you tell whether everyone involved was comfortable with the outcome?
- What worked well / what could have been changed

*Rationale: items of interest in terms of processes/areas where support needed may emerge organically. If everyone mentions something this could use up quite a bit of the meeting, actually.*

3. We have talked about quite a few ethical issues this morning/afternoon. Thank you that’s been really helpful.

**If it was up to you, what kinds of things would you put in place to support staff working through these sorts of issues?**

*As people make suggestions ask the group* - if they have had experience of doing this and how useful it was/ Why do you think it would be useful?

- Then we can introduce some probes around formal/informal; in working hours/outside working hours; facilitated / unfacilitated etc but I wonder whether these could be down with an online qual survey perhaps or in another FG designed to look in more detail at options?

Thank you very much for participating. I will now stop the recording.

If you would like more information or support with anything that we have covered today, please see the debrief information that we have emailed to you.

The transcript will be produced using an automated service, and we will then check for any identifying information which will be removed. It will then be analysed alongside our interviews with healthcare professionals. If you would like a copy of the final report, please contact [email address] and we will send you a copy when it is available
